# Role of Cytokines, Chemokines and IFN-γ^+^ IL-17^+^ Double-Positive CD4^+^ T Cells in Patients with Multiple Sclerosis

**DOI:** 10.3390/biomedicines10092062

**Published:** 2022-08-24

**Authors:** Marlos Aureliano Dias de Sousa, Chamberttan Souza Desidério, Jonatas da Silva Catarino, Rafael Obata Trevisan, Djalma Alexandre Alves da Silva, Vinicius Ferreira Resende Rocha, Weslley Guimarães Bovi, Rodolfo Pessato Timoteo, Renata Cristina Franzon Bonatti, Alex Eduardo da Silva, Alfredo Leboreiro Fernandez, Helioswilton Sales-Campos, Virmondes Rodrigues Junior, Marcos Vinicius da Silva, Carlo José Freire de Oliveira

**Affiliations:** 1 Department of Immunology, Microbiology and Parasitology, Federal University of Triângulo Mineiro, Uberaba 38025-180, MG, Brazil; 2 Department of Neurology, Federal University of Triângulo Mineiro, Uberaba 38025-180, MG, Brazil; 3 Program in Integrative Cell Signaling and Neurobiology of Metabolism, Department of Comparative Medicine, Yale University School of Medicine, New Haven, CT 06510, USA; 4 Institute of Tropical Pathology and Public Health, Federal University of Goiás, Goiania 74605-050, GO, Brazil; 5 Laboratory of Immunology and Bioinformatics, Universidade Federal do Triângulo Mineiro, Rua Vigário Carlos, 100, 8th Floor, Uberaba 38025-350, MG, Brazil

**Keywords:** Multiple sclerosis, cytokine, chemokine, double-positive CD4^+^ T cells

## Abstract

Multiple sclerosis is mediated by self-reactive myelin T and B cells that lead to axonal and myelin damage. The immune response in multiple sclerosis involves the participation of CD4^+^ T cells that produce cytokines and chemokines. This participation is important to find markers for the diagnosis and progression of the disease. In our work, we evaluated the profile of cytokines and chemokines, as well as the production of double positive CD4^+^ T cells for the production of IFNγ IL-17 in patients with multiple sclerosis, at different stages of the disease and undergoing different treatments. We found that relapsing–remitting patients had a significant increase in IL-12 production. About IL-5, its production showed significantly higher levels in secondarily progressive patients when compared to relapsing–remitting patients. IFN-γ production by PBMCs from secondarily progressive patients showed significantly higher levels. This group also had a higher percentage of CD4^+^ IFNγ^+^ IL-17^+^ T cells. The combination of changes in certain cytokines and chemokines together with the presence of IFNγ^+^ IL-17^+^ double positive lymphocytes can be used to better understand the clinical forms of the disease and its progression.

## 1. Introduction

Multiple sclerosis is an inflammatory, autoimmune, demyelinating, and long-lasting disease of the brain and spinal cord, characterized by the presence of multiple disruptions in central and peripheral self-tolerance. More specifically, the disease presents myelin-reactive T and B cells that induce myelin and axonal damage. Its pathogenesis is related to a combination of environmental, genetic, intestinal microbiota, and immune system disorders. Furthermore, this chronic disease leads to physical, biological, psychological, and social damage to affected individuals. MS is the leading cause of disability related to neurological diseases in young people and globally affects females in a 2:1 ratio [1]. The distribution varies according to the climatic aspects, where temperate countries have a higher incidence and prevalence than tropical countries; in a simplified way, the farther from the equator, i.e., the greater the latitude, the greater the prevalence. It has prevalence rates ranging from 2 per 100,000 in Asia to over 100 per 100,000 in Europe and North America. In Brazil, a country with a tropical climate and a highly heterogeneous population, the prevalence of MS is 18/100,000 inhabitants [2,3].

Clinically, MS is categorized as remitting relapse (RR), secondary progressive (SP), and primary progressive (PP) [4]. Regardless of the clinical form, its initial stage is followed by the exacerbated immune and inflammatory response, on the other hand, later stages are marked by reduced inflammation, noticeable neurodegeneration, and severe disability [1,5]. The direct and indirect costs of the treatment are high and are mainly related to the acquisition of disease-modifying therapy (DMT) available in the country and subsidized by the Federal Government of Brazil through the Ministry of Health [2,3]. Currently, DMTs are glatiramer acetate, interferon beta 1a, teriflunomide, azathioprine, methylprednisolone (only for the treatment of outbreaks), dimethyl fumarate, fingolimod, and natalizumab. Among them, the most used in Brazil are glatiramer acetate, interferon beta1a, and fingolimod [6].

The first description of the induction of cytokine production in MS patients dates back to 1976 when Degré et al. (1976) showed that the cerebrospinal fluid of MS patients had increased interferon (IFN) type 1 titers [7]. Leukocytes from the bloodstream of patients with the disease have been shown to produce greater amounts of tumor necrosis factor-alpha (TNF-α), interleukin 1-beta (IL1-β), and IFN-γ [8,9,10]. In 1995, it was shown that in active lesions of patients with MS there was an increase in IL-1, IL-2, IL-4, IL-10, TNF-α, tumor growth factor-beta (TGF-β), and IFN-γ [11]. Serum cytokines INF-γ, IL-4 [12], lymphotoxin, and IL-12 are generally increased in clinical exacerbation phases, while TGF-β and IL-10 are increased in remission [13]. In patients with the PP form, there was a decrease in the percentage of CD4^+^ T cells producing IL-2, TNF-α, and IL-13 and an increase in the percentage of CD8^+^ T cells producing IL-4 or IL-10 when compared to the RR and SP forms [14]. Furthermore, Kallaur et al. (2013), investigating the RR clinical form, demonstrated that the levels of IFN-γ, IL-6, IL-12, and IL-4 were higher than in healthy patients [15]. The consequent increase that occurs in the levels of IL-12 p40, TNF-α, and IL-1β and a decrease in the production of IL-10 in the RR, SP, or PP forms have also been demonstrated [16,17]. In peripheral blood, the frequency of IL-17^+^ or IFN-γ^+^ mononuclear cells were increased in patients with the RR and SP forms [18].

In addition to the importance of identifying key molecules in MS pathogenesis, exploring its origin is of paramount importance to understanding the contribution of different cell populations to disease outcomes. Independent populations of CD4+ T lymphocytes can produce IFN-γ or IL-17, cytokines with a known pathogenic role in MS [19,20]. As in MS, in general, Th cells are described as isolated producers of a certain pattern of cytokines, such as IL-17 and IFN-γ. However, in some infectious diseases such as malaria, toxoplasmosis, and Chagas disease, the contribution of dual cell populations that produce these cytokines in the pathophysiology of these diseases has been observed. Despite this, this type of population is still poorly explored in immune-mediated diseases such as MS. Thus, this study aimed to analyze the cytokine profile in MS patients treated with glatiramer acetate, interferon β 1a, and fingolimod, with the different clinical forms of the disease, as well as to identify a possible contribution of T CD4^+^ IFN-γ^+^ IL-17^+^ cells in MS pathophysiology.

## 2. Materials and Methods

### 2.1. Patients and Study Design

This is an observational and analytical study with patients diagnosed with MS (according to McDonald’s 2010 criteria) and followed up at the Neurology Clinic of the Federal University of Triângulo Mineiro (UFTM). Patients were included regardless of the clinical form or drug therapy used. Patients with MS (*n* = 30) were included in the study and compared to a control group composed of healthy individuals (healthy control *n* = 21). Participants in the healthy control group had a similar demographic profile to the MS patient group. All individuals enrolled in the study signed the Term of Consent after reading the Term of Clarification, answered a standardized questionnaire, and accepted to participate in the research. This research was approved by the Research Ethics Committee of the Federal University of Triângulo Mineiro (1.870.116; CAAE 61990816.3.0000.5154). All respondents were informed about the research protocol before enrolling in the investigation, following the Declaration of Helsinki.

### 2.2. Exclusion Criteria

The following criteria were used to exclude patients from this study: (a) patients who did not meet the McDonald 2010 criteria; (b) patients with fever; (c) or any reports of a previous diagnosis of autoimmune or infectious diseases (such as dengue, lupus erythematosus, rheumatoid arthritis, autoimmune nephropathies, liver diseases). The information was collected during the interview that preceded the blood collection and was confirmed with data from each patient’s medical record.

### 2.3. Blood Collection

Peripheral venous blood was collected in two 10 mL tubes containing heparin while the patients were at the UFTM Neurology Outpatient Clinic. One of the tubes was centrifuged and the plasma removed for later measurement of cytokines and chemokines. The other one was used for obtaining peripheral blood mononuclear cells (PBMC) as described below.

### 2.4. Isolation of Mononuclear Cells

Peripheral blood mononuclear cells (PBMC) were isolated by a Ficoll–Hypaque density gradient (GE Health Care, Uppsala, Sweden). Briefly, cells were centrifuged at 400× *g* for 30 min at 21 °C. The obtained PBMCs were resuspended in RPMI 1640 medium (GE) containing 50 mM Hepes (GIBCO, Grand Island, NY, USA), 5% inactivated fetal bovine serum (GIBCO, Grand Island, NY, USA), 2 mM L-glutamine (GIBCO, Grand Island, NY, USA), 40 μg/mL gentamicin (Neoquímica, Anápolis, State of Goiás, Brazil), 1 mL 2β-mercaptoethanol (Merck, Darmstadt, Germany), in the final concentration of 1 × 10^6^/mL and then cultured in 96-well plates (FALCON, San Jose, CA, USA) under stimulation with anti-CD3 for 24 h in an incubator with 5% CO_2_. All procedures were performed under sterile conditions, using a laminar flow hood. After 24 h of culture, the supernatant was collected to measure cytokines and the other part was frozen at −70 °C. In addition, cells were collected to evaluate intracellular cytokine production.

### 2.5. Production of Chemokines, Cytokines, and BDNF

Production of IL-1β, IL-2, IL-4, IL-5, IL-6, IL-8, IL-10, IL-12, IL-13, IL-15, IL-23, IL-33, CCL-10 (IP-10), CCL-11 (eotaxin-1), IFN-g, TNF-α, and brain-derived neurotrophic Factor (BDNF) were investigated in plasma using enzyme immunoassay (R&D Systems^®^, San Diego, CA, USA), following the manufacturer’s instructions and expressed in pg/mL, the material used was stored in a −80° freezer from collection and underwent two thawing cycles. The cytometric bead array (CBA) was used to measure cytokines and chemokines in the cell culture supernatant following the manufacturer’s instructions to address the production of the following chemokines CXCL8/IL-8, CCL5/RANTES, CXCL9/MIG, CCL2/MCP-1, and CXCL10/IP-10. The Human Th1/Th2/Th17 Cytokine Kit (BD Biosciences, San Jose, CA, USA) was also used, and the acquisition was performed on a BD FACSCanto™ flow cytometer (BD Immunocytometry Systems, BD Biosciences, San Jose, CA, USA). The concentration of the sample was estimated by comparing the fluorescence of PE obtained from the standard curve obtained by serial dilution of recombinant human chemokines and cytokines. Results were analyzed by 5-Parameter Logistic Regression in FCAP array software and expressed in pg/mL.

### 2.6. Characterization of Cytokine Double-Producers CD4^+^ T Lymphocytes

The PBMCs (2 × 10^6^ cells/mL) from MS patients, sick or healthy controls were stimulated or not during 24 h, with Anti-CD3 (1 mL/mL (BD Biosciences, San Jose, CA, USA) + Anti-CD28 (1 mL/mL) (BD Biosciences, San Jose, CA, USA). For analysis of surface markers and intracellular cytokines, PBMCs were first incubated with PBS-1X supplemented with 10% inactivated human serum (AB+) for 30 min. The cells were then labeled with antibodies directed to the following surface molecules: Anti-CD4-PE-Cy5 (BD Biosciences, San Jose, CA, USA) and Anti-CD69-PE (BD Biosciences, San Jose, CA, USA). The antibodies were chosen according to the convenience of the analysis to be performed later. The cells were then washed (400 g, 4 °C, 10 min), fixed, and permeabilized with 250 μL of Cytofix/Cytoperm (BD Biosciences, San Jose, CA, USA), for 30 min at 4 °C. Then, the cells were washed in Permwash (B BD Biosciences, San Jose, CA, USA) containing 10% fetal bovine serum (SIGMA, San Luis, MO, USA), and then labeled with antibodies to the following intracellular cytokines: IFN-γ-APC, IL-17-Alexa488, and IL-10-PE (BD Biosciences, San Jose, CA, USA). At the end of this period, the cells were again washed in Permwash and resuspended until the time of analysis on the flow cytometer. Event acquisition (50000 events/sample/tube) was performed on a FACSCalibur (BD Biosciences, San Jose, CA, USA) cytometer using the Cell Quest program (BD Biosciences, San Jose, CA, USA). Data analysis was performed using the FlowJo program (TREESTAR, Woodburn, OR, USA) from the individualization of the leukocyte population utilizing “Gates” established according to the size (FSC) and granularity (SSC) standards compatible with the lymphocyte population.

### 2.7. Statistical Analysis

Normal distribution and homogeneous variance were tested for all study variables. Then, the D’Agostino–Pearson test was used to assess normality. In cases of non-Gaussian distribution, the nonparametric Mann–Whitney test was applied. Multiple comparisons regarding median values for more than two groups were performed using the nonparametric Kruskal–Wallis test followed by Dunn’s test. Differences were considered statistically significant when the probability of their occurrence was less than *p* < 0.05 (5%). Statistical analysis was performed using GraphPad Prism software (GraphPad Software 8.0, La Jolla, CA, USA). To achieve the correlation analysis after finding the non-Gaussian distribution, Spearman’s correlation analysis was applied, where the correlation was considered significant when *p* < 0.05. In addition to using the Jamovi (Jamovi 2.5.1, Sydney, Austrália) software to prepare tables and evaluate the analysis of qualitative variables.

## 3. Results

### 3.1. Clinical and Epidemiological Profile of Individuals Enrolled in the Study

Healthy volunteers were mainly composed of women (95%) aged between 21 and 57 years (mean 40.76 ± 8.91), as shown in Table 1. The group of patients with MS had the following distribution: 21 with the RR form (70%), 6 with SP (20%), 1 PP (3.33%), and 2 with isolated clinical syndrome (6.66%) (see Table 1). The mean age of patients with MS was 41.9 years (±10.9) and most were women (76%).

Patients with MS were classified according to clinical form and in Table 2, the treatment times in months for each group were presented. The SP group had a significantly longer treatment time compared to the other groups.

When classified by the treatment used at the time of collection. The drug most used by patients with MS was interferon Β1-a (43.3%), regardless of the clinical form, followed by the use of glatiramer acetate. Table 3 shows the number of patients using each drug, in addition to data on the average time of treatment with each of these drugs.

### 3.2. The Recurrent Relapsing Form of MS Demonstrates an Increase in the Production of Pro-Inflammatory Chemokines and Cytokines (IP-10, IL-12, and Eotaxin)

**Figure 1:**
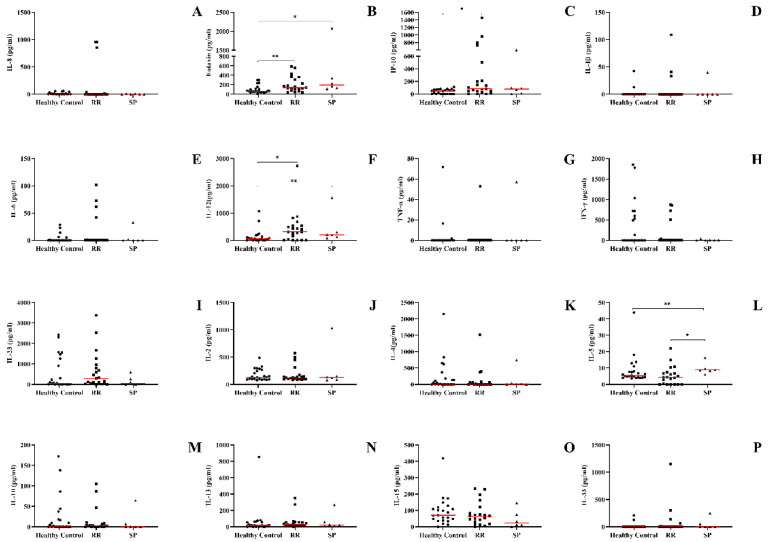
Concentration of cytokines and chemokines in the plasma of patients with multiple sclerosis according to the clinical form and compared to the group of healthy individuals and other neurological diseases obtained by enzyme-linked immunosorbent assay. (**A**) IL-8, (**B**) CCL-11/eotaxin-1, (**C**) CXCL-10/IP-10, (**D**) IL-1β, (**E**) IL-6, (**F**) IL-12, (**G**) TNF-α, (**H**) IFN-γ, (**I**) IL-23, (**J**) IL-2, (**K**) IL-4, (**L**) IL-5, (**M**) IL-10, (**N**) IL-13, (**O**) IL-15 e (**P**) IL-33. Healthy control, RR (relapse–remitting multiple sclerosis), SP (secondary progressive), and sick control (other neurological diseases). Rows represent the median. Statistical analysis was performed using the Kruskal–Wallis test with Dunn’s post-test, where * and ** represent *p* < 0.05.

To evaluate the profile of cytokines and chemokines of MS patients according to the clinical form, blood samples were collected. Cytokines and chemokines involved in both innate and adaptive immune responses were assayed. MS patients with the RR form, when compared to healthy individuals, had a higher concentration of IP-10 and IL-12 (Figure 1C,F). Regardless of the clinical form or the occurrence of other neurological disorders, eotaxin/CCL11 levels were higher when compared to the healthy controls (Figure 1B). Finally, patients with the SP clinical form had a higher concentration of IL-5 in the plasma when compared to the individuals with the RR form (Figure 1L). No differences were observed regarding the production of IL-1β, IL-6, IL-8, IL-4, IL-13, IFN-γ, IL-23, IL-2, TNF-α, IL-15, IL-33, and IL-10 (Figure 1A,D,E,G,H–K,M–P).

### 3.3. Secondary Progressive Form of MS Positively Regulates Levels of IL-4, IL-17, and IFN-γ in the Supernatant of PBMCs from MS Patients

**Figure 2:**
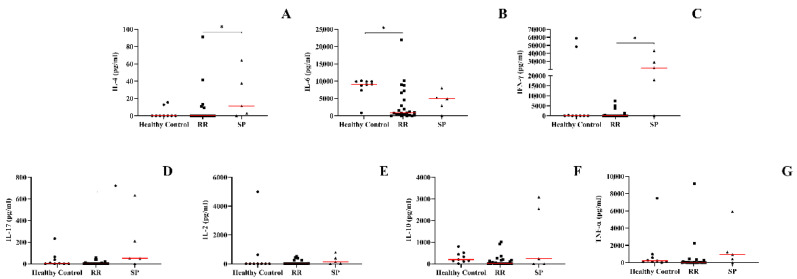
Concentration of cytokines through cultures of PBMCs derived from peripheral blood of the patient groups evaluated by CBA (cytometric bead array): Healthy control, RR (patients with relapse–remitting multiple sclerosis), SP (patients with secondary progressive multiple sclerosis). (**A**) IL-2, (**B**) IL-4, (**C**) IFN-γ, (**D**) IL-10, (**E**) IL-17, (**F**) TNF-α, and (**G**) IL-6. Rows represent the median. Statistical analysis was performed using the Kruskal–Wallis test with Dunn’s post-test, where * *p* < 0.05.

After evaluating the production of cytokines and chemokines in the plasma of patients with MS, we sought to assess whether unstimulated PBMCs were modulated to produce molecules involved in the pathogenesis of the disease. The SP form was associated with higher levels of IL-4 and IL-17 when compared to the RR form, when compared to the healthy control, we did not observe statistically significant differences (Figure 2A,B). The amount of IFN-γ was higher in the SP group when compared to healthy individuals, when compared to patients with the SP form, no significant differences were found in the levels of cytokines (Figure 2C). Healthy individuals produced greater amounts of IL-6 when compared to the EMRR group (Figure 2G). IL-2, IL-10, and TNF-α concentrations did not show statistically significant concentrations between groups (Figure 2D–F).

### 3.4. IL-12, Eotaxin, and IP-10 Were Increased in Patients with a Higher Degree on the EDSS Scale, While IL-5 Was Increased in Patients with the SP Form

**Figure 3:**
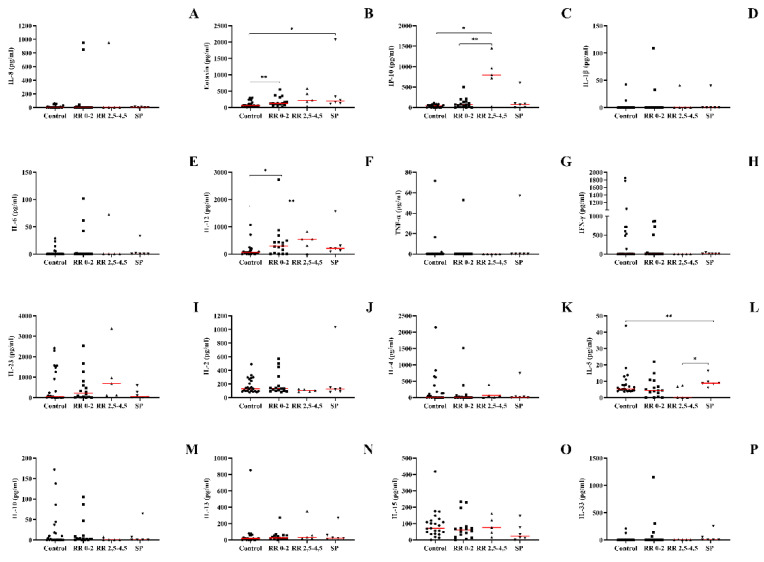
Concentration of cytokines and chemokines in the plasma of patients with multiple sclerosis according to clinical form and EDSS score compared to healthy individuals obtained by enzyme-linked immunosorbent assay. (**A**) IL-8, (**B**) CCL-11/eotaxin-1, (**C**) CXCL-10/IP-10, (**D**) IL-1β, (**E**) IL-6, (**F**) IL-12, (**G**) TNF-α, (**H**) IFN-γ, (**I**) IL-23, (**J**) IL-2, (**K**) IL-4, (**L**) IL-5, (**M**) IL-10, (**N**) IL-13, (**O**) IL-15, and (**P**) IL-33. Healthy control, RR (relapse–remitting multiple sclerosis) subdivided: EDSS 0–2 and EDSS 2.5–4.5; SP (secondary progressive multiple sclerosis). Rows represent the median. Statistical analysis was performed using the Kruskal–Wallis test with Dunn’s post-test, where * and ** represent *p* < 0.05.

After identifying the changes in the concentrations of cytokines and chemokines according to the clinical forms, we sought to understand whether the severity of the disease could also be related to the levels of these molecules in the plasma. Therefore, we demonstrate the levels of cytokines through the EDSS severity scale. When evaluating the production of IL-12, we found that both patients with the RR form with a lower score on the scale, and patients with the SP form, showed an increase in the production of IL-12 when compared to the healthy control group; other groups were not found to have significant differences (Figure 3F). Patients with the RR form showed an increase in the production of IP-10 when compared to the healthy control group, with no other significant differences found (Figure 3C). The associated pattern of eotaxin production was the same as that of IL-12, where both patients with the RR form with a lower score on the EDSS scale and patients with the SP form showed an increase in eotaxin production when compared to the healthy control group; about the other comparisons, no significant differences were verified (Figure 3B). IL-5 production was significantly increased in patients with the SP form when compared to the group with the highest EDSS score and with the RR form, the other comparisons showed no significant difference (Figure 3L). The following cytokines did not show significant differences with any analysis performed: IL-1, IL-6, IL-8, IL-4, IL-13, IFN-γ, IL-23, IL-2, TNF-α, IL-15, IL-10, and IL-33.

**Figure 4:**
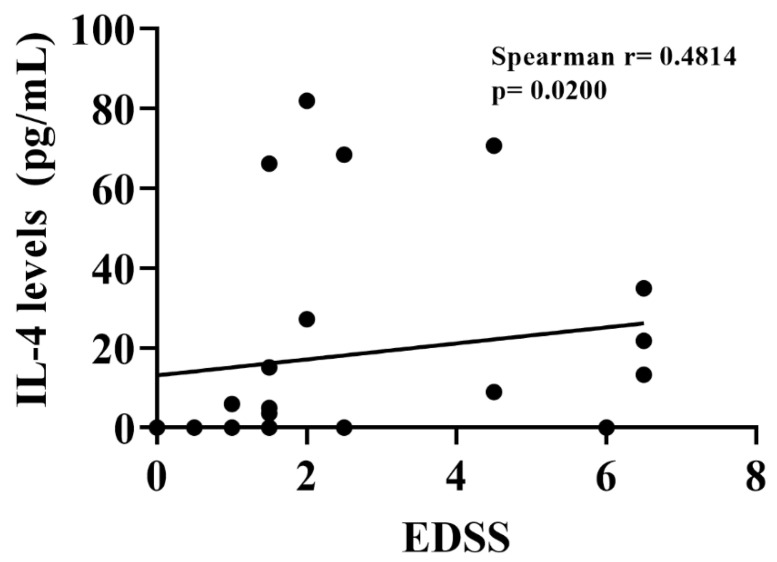
Dispersion graphic between Expanded Disability Status Scale (EDSS) and plasma interleukin 4 (IL-4) levels in patients with relapsing–remitting multiple sclerosis (Spearman’s rank correlation, *r* = 0.4814 with *p* = 0.0200).

To verify whether there was a correlation between disease severity, a Spearman correlation test was performed between the EDSS scale score and the following cytokines: IL-1β, IL-2, IL-4, IL-5, IL-6, IL-8, IL-10, IL-12, IL-13, IL-15, IL-23, IL-33, TNF-α, and IFN-γ, in addition to the correlation with the chemokines IP-10 and eotaxin (Table 4). IL-4 was the only cytokine with a significant correlation, showing a positive correlation with EDSS scores, the Spearman’s r and *p* values presented are from the analysis performed after the outliers were removed. (*r* = 0.4115, *p* = 0.0330) (Figure 4).

### 3.5. Cytokine and Chemokine Profile in the Plasma of Patients with MS Changes According to Treatment

**Figure 5:**
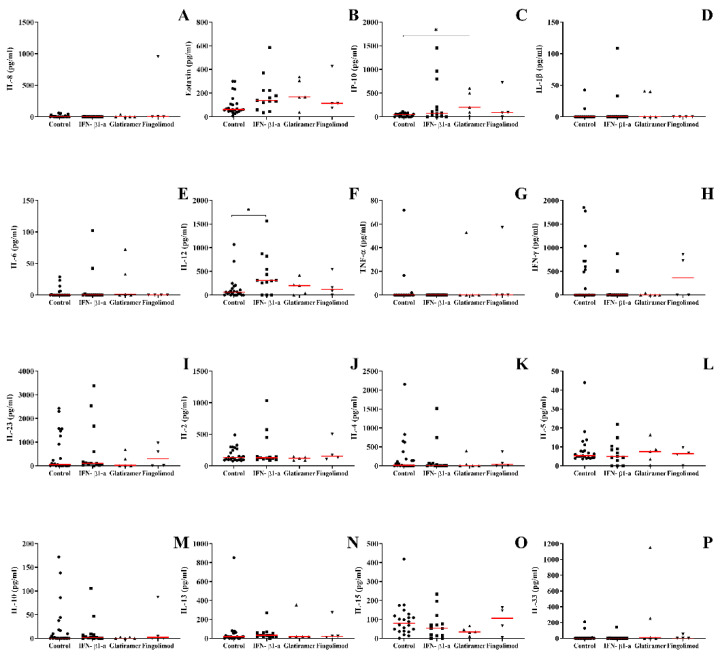
Concentration of cytokines and chemokines in patients with multiple sclerosis separated into groups according to the treatment used obtained by enzyme-linked immunosorbent assay: (**A**) IL-8, (**B**) CCL-11/eotaxin-1, (**C**) CXCL-10/IP-10, (**D**) IL-1β, (**E**) IL-6, (**F**) IL-12, (**G**) TNF-α, (**H**) IFN-γ, (**I**) IL-23, (**J**) IL-2, (**K**) IL-4, (**L**) IL-5, (**M**) IL-10, (**N**) IL-13, (**O**) IL-15, and (**P**) IL-33. Healthy control; IFN-β1-a = relapse–remitting multiple sclerosis patients treated with interferon β 1-a; glatiramer acetate = relapse–remitting multiple sclerosis patients treated with glatiramer acetate; fingolimod = relapse–remitting multiple sclerosis patients treated with fingolimod. Rows represent the median. Statistical analysis was performed using the Kruskal–Wallis test with Dunn’s post-test, where * represents *p* < 0.05.

Once we identified changes in plasma concentrations of cytokines and chemokines according to the clinical forms and severity of MS, we sought to investigate the impact of different pharmacological therapies in this scenario. In this analysis, we found that patients, regardless of the clinical form, who are being treated with IFN-b1 had increased levels of IL-12 production when compared to individuals in the control group, the other comparisons showed no significant difference (Figure 5A). Both the treatment with IFN-b1 and glatiramer acetate were increased when compared to the healthy control group; about the other comparisons, we found no significant difference (Figure 5B). The production of IP-10 was significantly increased only in patients treated with glatiramer acetate. No changes were observed in patients treated with fingolimod. The concentration of IL-1, IL-6, IL-8, IL-4, IL-13, IFN-γ, IL-23, IL-2, TNF-α, IL-5, and IL- and IL-33 did not show significant differences when all groups were compared to each other (Figure 5).

### 3.6. The Production of BDNF in the Plasma of Patients with MS Was Not Altered According to the Clinical Form or Treatment Used

**Figure 6:**
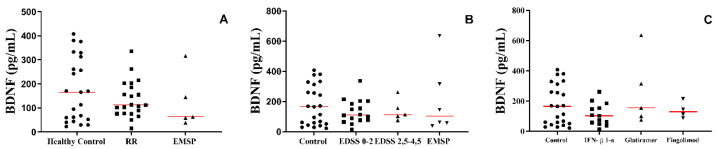
Plasma’s BDNF protein level in different scenarios. (**A**) Between the groups: healthy control, RR group (patients with relapse–remitting multiple sclerosis), EMSP group (patients with secondary progressive multiple sclerosis), and patient control (other neurological diseases). (**B**) Between the groups: healthy control, RR group (patients with relapse–remitting multiple sclerosis) divided according to EDSS scale: 0–2 and 2.5–4.5; EMSP group (patients with secondary progressive multiple sclerosis). (**C**) Between the groups: healthy control and RR group divided according to the treatment: IFN-β1-a = interferon Β 1-a, glatiramer acetate, and fingolimod. Rows represent the median. We used the Kruskal–Wallis test with Dunn’s post-test.

Regardless of the group, the clinical form, the severity of the disease, or the targeted treatment for each scenario, no differences were observed in the production of BDNF (Figure 6).

### 3.7. PBMCs from Patients with the Secondarily Progressive Form Shows an Increase in the Percentage of IFN-γ- and IL-17–Producing Dual Auxiliary T Lymphocytes (TCD4^+^IFN-γ^+^IL-17^+^)

**Figure 7:**
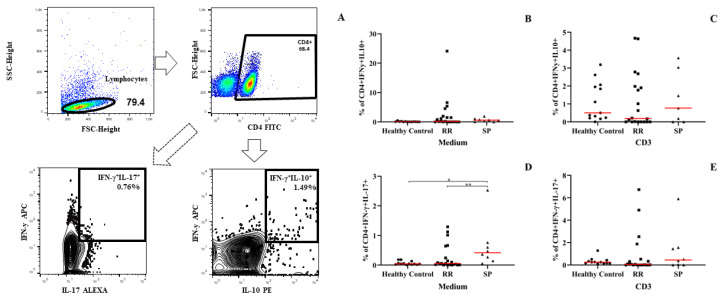
Flow cytometry evaluation of double CD4+ T cells producing cytokines in patients with multiple sclerosis. (**A**) Schematic representation of the gating strategy for the acquisition of flow cytometry data for the culture of PMBCs, with the gates proposals for CD4^+^IFN-γ^+^IL-10^+^ lymphocytes and CD4^+^IFNγ^+^IL17^+^. (**B**) The percentage of CD4^+^IFNγ^+^IL10^+^ T lymphocytes in the cells in the absence of stimulation. (**C**) The percentage of CD4^+^IFNγ^+^IL10^+^ lymphocytes in cells stimulated with anti-CD3. (**D**) The percentage of CD4^+^IFNγ^+^IL17^+^ T lymphocytes in cells without stimulation. (**E**) The percentage of CD4^+^IFNγ^+^IL17^+^ T lymphocytes in anti-CD3-stimulated cells. Statistical analysis was performed using the Kruskal–Wallis test with Dunn’s post-test, where * and ** represents *p* < 0.05.

After evaluating the production of cytokines and chemokines in the different situations presented, we investigated the essential role that IFN-γ- and IL-17-producing dual helper t lymphocytes can play in the pathophysiology of MS. We evaluated the difference in different clinical forms. Regarding the analysis of IFN-γ-and IL-17-producing dual helper lymphocytes, we found that patients with the secondarily progressive form had a significantly higher percentage of CD4^+^ T lymphocytes simultaneously producing IFN-γ and IL-17 when compared to healthy individuals and patients with the RR form (Figure 7D). This increase was only verified before stimulation with CD3; when stimulated with CD3, we did not observe any significant difference (Figure 7E). Regarding the percentage of TCD4^+^ lymphocytes that produce IL-10 and IL-17, the PBMCs from patients with MS stimulated or not with anti-CD3 did not show alterations (Figure 7B,C). These results assume that this cell type may be closely related to the clinical form and a consequent greater severity of the disease. Since we observed this cell type in a more severe form and where patients had a higher score on the EDSS severity scale.

## 4. Discussion

The findings of our study suggest that there are molecules that can be effectively related to a particular clinical form of MS, in addition to molecules that can be closely related to disease severity, being correlated with the increase in severity measured by the EDSS scale. We also brought up a point of extreme importance and still little investigated, which is the presence and action of double helper lymphocytes that produce IFN-γ and IL-17, which were present and increased in patients with more severe forms of MS. In our study, we found that patients with the RR form of MS had higher levels of IL-12 in comparison to healthy individuals, even when the RR group was subdivided using the EDSS scale as a severity criterion. This result is in line with the literature that demonstrates that serum levels of IL-12 are elevated in patients with multiple sclerosis and are also related to higher levels in the active form of the disease, which explains the higher level of IL-12 presented by patients with the RR form [21].

A higher production of CXCL10/IP-10 was also observed in patients with the RR form when compared to their healthy counterparts. This was observed also in patients with the RR form with the highest score on the EDSS scale. Considering the role of IP-10 as a co-stimulatory molecule in the stimulation of IFN-γ production, it can be assumed that increased levels of IP-10 and IL-12 may have a cause-and-effect relationship and be a possible clinical marker to be explored in further research. To discover a biological marker for the early diagnosis of MS or in an attempt to clinically stratify the disease, researchers have already demonstrated that concentrations of IP-10—combined with the combination with IL-7—seem to serve to rule out the clinical form of PP [22].

Elevated levels of the chemokine eotaxin (CCL11) occurred in the RR and SP groups compared to the healthy subjects. When the groups were subdivided by the EDSS scale, plasma eotaxin levels remained high in the SP group and in the RR group with EDSS 2.5–4.5. When we tried to assess the difference between the RR and SP groups, there was no statistical significance, although we considered that there was a tendency for eotaxin concentrations to be higher in the SP group. Eotaxin is highly expressed at the site of brain injury and is closely related to the neurodegeneration process that occurs in multiple sclerosis [23]. As demonstrated in our findings, eotaxin is associated with the evolution of the disease course, researchers have demonstrated this relationship in experimental autoimmune encephalomyelitis induced by myelin oligodendrocyte glycoprotein (MOG) (EAE), one of the most used animal models to simulate multiple [24]. Data similar to ours showed that the association of four markers—HGF, eotaxin/CCL11, EGF, and MIP-1β/CCL4—showed a strong correlation to detecting progressive forms of MS [25].

In our study, IL-5 was increased in patients with the SP form when compared to the RR group, even with the division by the EDSS scale, the group with the SP form showed a significant increase concerning patients with the EDSS scale of 2.5–4.5. This possible increase in IL-5 could mean the use of this marker as a specific marker for the SP form of MS. Since the work of H. Ochi and collaborators showed a correlation between the production of IL-13 and IL-5 in the RR phase of MS, they found that there is an increase in IL-13 but no increase in the production of IL-5 produced by TCD4^+^ and TCD8^+^ lymphocytes compared to the healthy controls, indicating that IL-5 would not be related to the RR form of MS [26].

Although there were no changes in the production of cytokine IFN-γ in the plasma of MS patients, when we evaluated the production of this cytokine in the supernatant of PBMCs, there was an increase, particularly in patients with SP. These data suggest that increased IFN-γ (the main cytokine to determine the differentiation of naive CD4^+^ T cells in Th1 lymphocytes) may be related to clinical forms of patients with a higher degree of disability. The increase in the concentration of IFN-γ is implicated as a determinant factor for the pathophysiological process of MS and experimental autoimmune encephalitis (EAE), an experimental model of MS [27,28]. Some researchers have attempted to treat MS with the use of IFN-γ for 18 patients, but 7 of them had exacerbation of the disease and, in addition, found an increase in the proliferation of peripheral blood leukocytes and an increase in activated NK cells [29].

Another study showed that the elevation of IFN-γ production by peripheral lymphoid cells is related to the onset of symptoms in EAE and that increased IFN-γ secretion by mononuclear cells in the central nervous system of individuals with MS was higher than in healthy individuals [27]. Although the increased concentration of IFN-γ is related to the increase in inflammatory activity and the aggravation of EAE and MS, other studies have shown a much more comprehensive action, including a protective role in these cases [28,30]. It is noted, therefore, that there is a great complexity involved in the relationship between cytokines and their role in MS. When we considered the analysis of individuals with SP, we found that IL-4, IL-5, and IL-17 cytokines were significantly higher in this group when compared to individuals with the RR form. Two patterns of the immune response are clearly defined here: one Th2 (which synthesizes IL-4 and IL-5) and another Th17 (determined by IL-17 production). Thus, patients with the SP form evaluated in our study had a Th1, Th2, and Th17 immune response pattern. The concomitance of these three immune response patterns in MS patients is unprecedented, mainly because it is a specific clinical form characterized by a neurodegenerative pattern that is much more evident than neuroinflammatory. There is evidence obtained through experimental work on tissue models for autoimmunity that the pathway of differentiation and production of the Th17 lineage is completely distinct and independent of the Th1 and Th2 lineages [31]. Because it is evidence from studies in animal models, it is exceedingly difficult to simply transpose this information to humans.

More data obtained in our experiments that deserve special attention refers to the high concentration of CD4^+^ T-cells producing both IFN-γ and IL-17 cytokines in patients with SP. The amount of IFN-γ^+^IL-17^+^CD4^+^ T cells was higher in patients with SP when compared to individuals with RR or compared to the group of healthy individuals. The amount of IFN-γ^+^IL-17^+^CD4^+^ T cells in SP individuals may be one of the reasons for these patients to maintain three different types of immune patterns: Th1, Th2, and Th17. Our results show differences in the production of cytokines and chemokines in the plasma of patients at different stages of the disease, showing the production of pro-inflammatory molecules and cells in more severe stages of the disease, our data are complemented by the work of Donninelli and collaborators (2017) in who observed that several soluble factors are produced in the CSF of patients with MS, which the authors divide into two groups that have pro- and anti-inflammatory functions, showing different productions in patients with different stages of the disease, being related to EDSS. Where patients with more severe disabilities have an imbalance between the production of pro- and anti-inflammatory factors [32]. These findings cited above appear to be a major finding and may correlate with a clinical form of MS patients.

## 5. Conclusions

Although our results are promising to unravel some nuances of the immune response in patients with multiple sclerosis, we must highlight some limitations found, several in common when it comes to clinical research: despite our data presenting significant results, we are aware that we had a considered small sample; in addition, even with our data representing patients treated in a macro-region with approximately 26 municipalities, a multicenter study would give better quality to the work. From the point of view of understanding the disease itself and its pathophysiology, the presence of CIS patients (isolated clinical syndrome) would also prove to be important to verify the disease situation without the influence of treatment.

Given the aforementioned limitations and the difficulties in establishing a specific marker for the diagnosis of MS and markers that can be used to verify the progression of the disease and verify the effectiveness of the treatment, our study presents excellent possibilities, as there were markers that were expressed in greater amounts at a certain stage (form) of the disease. For example, IL-12, IP-10, and eotaxin showed higher expressions in patients who had the RR form of the disease. IL-5, on the other hand, showed an increase in its production in patients with the SP stage of the disease, that is, we have potential targets for the follow-up of patients with different forms of the disease, but further studies are needed to confirm this capacity of these markers, and for studies that focus on the early diagnosis of the disease. The cytokine and chemokine profile of MS patients seems to be a promising alternative for the development of diagnostic tools, evaluating the effectiveness of treatment or phenotypic change. Elevated plasma levels of IL-12 and IP-10 suggest indicating groups of patients with more inflammatory characteristics. Eotaxin, on the other hand, may be more strongly related to progressive forms of MS, which are known to be less inflammatory, but with a worse degree of functional impairment than the RR type.

## Figures and Tables

**Table 1:** Clinical-demographic data.

	Gender	Clinical Form	N	Mean	Median	SD	Minimum	Maximum
Age (year)	F	CIS	1	27.0	27	NaN	27	27
		HC	20	41.1	43	10.6	21	57
		RR	17	40.1	39	11.6	24	62
		SP	5	52.2	57	14.3	36	67
	M	CIS	1	37.0	37	NaN	37	37
		HC	1	34.0	34	NaN	34	34
		PP	1	39.0	39	NaN	39	39
		RR	4	32.8	33.0	11.5	19	46
		SP	1	60.0	60	NaN	60	60

Female (*n* = 23; 76.66%); M = male (*n* = 7; 23.33%); HC = healthy control RR = relapse–remitting multiple sclerosis; PP = primary progressive multiple sclerosis; SP = secondary progressive multiple sclerosis; CIS = clinical isolated syndrome. SD = standard deviation.

**Table 2:** Distribution of patients by clinical forms with treatment time.

	Clinical Form	N	Mean	Median	SD	Minimum	Maximum
Treatment time (Months)	CIS	2	1.50	1.50	2.12	0	3
	PP	1	21.00	21	NaN	21	21
	RR	21	48.05	31	52.34	0	162
	SP	6	106.17	102.50	67.99	24	206

Treatment time in months. RR = relapse–remitting multiple sclerosis; PP = primary progressive multiple sclerosis; SP = secondary progressive multiple sclerosis; CIS = clinical isolated syndrome. SD = standard deviation.

**Table 3:** Distribution of patients by treatment type with treatment time.

	Treatment type	N	Mean	Median	SD	Minimum	Maximum
Treatment time (months)	Untreated	3	0.00	0	0.00	0	0
	IFN-β 1a	14	83.57	82.0	65.49	2	206
	Glatiramer Acetate	7	56.57	45	51.95	3	156
	Dimethyl Fumarate	1	5.00	5	NaN	5	5
	Fingolimod (FTY720)	4	22.50	24.0	8.35	11	31
	Corticosteroids	1	9.00	9	NaN	9	9

Treatment time in months. Treatment types: DMT = dimethyl fumarate; FTY720 = fingolimod; GA = glatiramer acetate; IFN-β 1a = interferon β 1a; Corticosteroids = methylprednisolone.

**Table 4:** Correlation table between cytokines and chemokines with the Expanded Disability Status Scale.

Cytokine	R Spearman	*p* Value	*p* Value Summary
IL-1β	0.05285	0.7935	ns
IL-2	−0.2934	0.1374	ns
IL-4	0.4814	0.0200	*
IL-5	0.3669	0.0597	ns
IL-6	−0.05699	0.7777	ns
IL-8	−0.05260	0.7944	ns
IL-10	−0.3281	0.0947	ns
IL-12	0.1336	0.5066	ns
IL-13	0.1654	0.4096	ns
IL-15	−0.03531	0.8612	ns
IL-23	−0.005240	0.9793	ns
IL-33	−0.09314	0.6440	ns
EOTAXIN	0.1187	0.5555	ns
IP-10	−0.08088	0.6884	ns
IFN-γ	0.04964	0.8058	ns
TNF-α	−0.05021	0.8036	ns
BDNF	0.001696	0.9933	ns

Spearman’s R-values and the *p*-value is shown, where * *p* < 0.05.

## Data Availability

The data presented in this study are available upon request from the corresponding author. The data are not publicly available due to the fact that there are data from medical records that, even with TCLE, are only available if requested, also because they are not yet published data.

## References

[1] Reich D.S., Lucchinetti C.F., Calabresi P.A. (2018). Multiple Sclerosis. N. Engl. J. Med..

[2] da Gama Pereira A.B., Sampaio Lacativa M.C., da Costa Pereira F.F., Papais Alvarenga R.M. (2015). Prevalence of multiple sclerosis in Brazil: A systematic review. Mult. Scler. Relat. Disord..

[3] Kobelt G., Teich V., Cavalcanti M., Canzonieri A.M. (2019). Burden and cost of multiple sclerosis in Brazil. PLoS ONE.

[4] Lublin F.D., Reingold S.C., Cohen J.A., Cutter G.R., Sorensen P.S., Thompson A.J., Wolinsky J.S., Balcer L.J., Banwell B., Barkhof F. (2014). Defining the clinical course of multiple sclerosis: The 2013 revisions. Neurology.

[5] Dendrou C.A., Fugger L., Friese M.A. (2015). Immunopathology of multiple sclerosis. Nat. Rev. Immunol..

[6] (2001). Brasil. Ministério Da Saúde Secretaria De Atenção à Saúde Secretaria De Ciência, Tecnologia e Insumos Estratégicos. https://bvsms.saude.gov.br/bvs/saudelegis/sas/2018/poc0011_09_04_2018.html.

[7] Degre M., Dahl H., Vandvik B. (1976). Interferon in the serum and cerebrospinal fluid in patients with multiple sclerosis and other neurological disorders. Acta Neurol. Scand..

[8] Chofflon M., Juillard C., Juillard P., Gauthier G., Grau G.E. (1992). Tumor necrosis factor alpha production as a possible predictor of relapse in patients with multiple sclerosis. Eur. Cytokine Netw..

[9] Huang W.X., Huang P., Link H., Hillert J. (1999). Cytokine analysis in multiple sclerosis by competitive RT–PCR: A decreased expression of IL-10 and an increased expression of TNF-alpha in chronic progression. Mult. Scler..

[10] Kahl K.G., Kruse N., Faller H., Weiss H., Rieckmann P. (2002). Expression of tumor necrosis factor-alpha and interferon-gamma mRNA in blood cells correlates with depression scores during an acute attack in patients with multiple sclerosis. Psychoneuroendocrinology.

[11] Cannella B., Raine C.S. (1995). The adhesion molecule and cytokine profile of multiple sclerosis lesions. Ann. Neurol..

[12] Link J., Soderstrom M., Olsson T., Hojeberg B., Ljungdahl A., Link H. (1994). Increased transforming growth factor-beta, interleukin-4, and interferon-gamma in multiple sclerosis. Ann. Neurol..

[13] Navikas V., Link H. (1996). Review: Cytokines and the pathogenesis of multiple sclerosis. J. Neurosci. Res..

[14] Killestein J., Den Drijver B.F., Van der Graaff W.L., Uitdehaag B.M., Polman C.H., Van Lier R.A. (2001). Intracellular cytokine profile in T-cell subsets of multiple sclerosis patients: Different features in primary progressive disease. Mult. Scler..

[15] Kallaur A.P., Oliveira S.R., Colado Simao A.N., Delicato de Almeida E.R., Kaminami Morimoto H., Lopes J., de Carvalho Jennings Pereira W.L., Marques Andrade R., Muliterno Pelegrino L., Donizete Borelli S. (2013). Cytokine profile in r multiple sclerosis patients and the association between progression and activity of the disease. Mol. Med. Rep..

[16] Alatab S., Maghbooli Z., Hossein-Nezhad A., Khosrofar M., Mokhtari F. (2011). Cytokine profile, Foxp3 and nuclear factor-kB ligand levels in multiple sclerosis subtypes. Minerva Med..

[17] Soldan S.S., Alvarez Retuerto A.I., Sicotte N.L., Voskuhl R.R. (2004). Dysregulation of IL-10 and IL-12p40 in secondary progressive multiple sclerosis. J. Neuroimmunol..

[18] Huber A.K., Wang L., Han P., Zhang X., Ekholm S., Srinivasan A., Irani D.N., Segal B.M. (2014). Dysregulation of the IL-23/IL-17 axis and myeloid factors in secondary progressive MS. Neurology.

[19] Babaloo Z., Aliparasti M.R., Babaiea F., Almasi S., Baradaran B., Farhoudi M. (2015). The role of Th17 cells in patients with relapsing-remitting multiple sclerosis: Interleukin-17A and interleukin-17F serum levels. Immunol. Lett..

[20] Kallaur A.P., Oliveira S.R., Simao A.N.C., Alfieri D.F., Flauzino T., Lopes J., de Carvalho Jennings Pereira W.L., de Meleck Proenca C., Borelli S.D., Kaimen-Maciel D.R. (2017). Cytokine Profile in Patients with Progressive Multiple Sclerosis and Its Association with Disease Progression and Disability. Mol. Neurobiol..

[21] Comabella M., Balashov K., Issazadeh S., Smith D., Weiner H.L., Khoury S.J. (1998). Elevated interleukin-12 in progressive multiple sclerosis correlates with disease activity and is normalized by pulse cyclophosphamide therapy. J. Clin. Investig..

[22] Fernandez-Paredes L., Casrouge A., Decalf J., de Andres C., Villar L.M., Perez de Diego R., Alonso B., Alvarez Cermeno J.C., Arroyo R., Tejera-Alhambra M. (2017). Multimarker risk stratification approach at multiple sclerosis onset. Clin. Immunol..

[23] Dhaiban S., Al-Ani M., Elemam N.M., Maghazachi A.A. (2020). Targeting Chemokines and Chemokine Receptors in Multiple Sclerosis and Experimental Autoimmune Encephalomyelitis. J. Inflamm. Res..

[24] Adzemovic M.Z., Ockinger J., Zeitelhofer M., Hochmeister S., Beyeen A.D., Paulson A., Gillett A., Thessen Hedreul M., Covacu R., Lassmann H. (2012). Expression of Ccl11 associates with immune response modulation and protection against neuroinflammation in rats. PLoS ONE.

[25] Tejera-Alhambra M., Casrouge A., de Andres C., Seyfferth A., Ramos-Medina R., Alonso B., Vega J., Fernandez-Paredes L., Albert M.L., Sanchez-Ramon S. (2015). Plasma biomarkers discriminate clinical forms of multiple sclerosis. PLoS ONE.

[26] Ochi H., Osoegawa M., Wu X.M., Minohara M., Horiuchi I., Murai H., Furuya H., Kira J. (2002). Increased IL-13 but not IL-5 production by CD4-positive T cells and CD8-positive T cells in multiple sclerosis during relapse phase. J. Neurol. Sci..

[27] Olsson T. (1992). Cytokines in neuroinflammatory disease: Role of myelin autoreactive T cell production of interferon-gamma. J. Neuroimmunol..

[28] Sanvito L., Constantinescu C.S., Gran B., Hart B.A. (2010). The multifaceted role of interferon-γ in central nervous system autoimmune demyelination. Open Autoimmun. J..

[29] Panitch H.S., Hirsch R.L., Schindler J., Johnson K.P. (1987). Treatment of multiple sclerosis with gamma interferon: Exacerbations associated with activation of the immune system. Neurology.

[30] Arellano G., Ottum P.A., Reyes L.I., Burgos P.I., Naves R. (2015). Stage-Specific Role of Interferon-Gamma in Experimental Autoimmune Encephalomyelitis and Multiple Sclerosis. Front. Immunol..

[31] Harrington L.E., Hatton R.D., Mangan P.R., Turner H., Murphy T.L., Murphy K.M., Weaver C.T. (2005). Interleukin 17-producing CD4+ effector T cells develop via a lineage distinct from the T helper type 1 and 2 lineages. Nat. Immunol..

[32] Donninelli G., Studer V., Brambilla L., Zecca C., Peluso D., Laroni A., Michelis D., Mantegazza R., Confalonieri P., Volpe E. (2021). Immune Soluble Factors in the Cere-brospinal Fluid of Progressive Multiple Sclerosis Patients Segregate into Two Groups. Front. Immunol..

